# Low toxicity and high immunogenicity of an inactivated vaccine candidate against COVID-19 in different animal models

**DOI:** 10.1080/22221751.2020.1852059

**Published:** 2020-12-10

**Authors:** Ze-Jun Wang, Hua-Jun Zhang, Jia Lu, Kang-Wei Xu, Cheng Peng, Jing Guo, Xiao-Xiao Gao, Xin Wan, Wen-Hui Wang, Chao Shan, Su-Cai Zhang, Jie Wu, An-Na Yang, Yan Zhu, Ao Xiao, Lei Zhang, Lie Fu, Hao-Rui Si, Qian Cai, Xing-Lou Yang, Lei You, Yan-Ping Zhou, Jing Liu, De-Qing Pang, Wei-Ping Jin, Xiao-Yu Zhang, Sheng-Li Meng, Yun-Xia Sun, Ulrich Desselberger, Jun-Zhi Wang, Xin-Guo Li, Kai Duan, Chang-Gui Li, Miao Xu, Zheng-Li Shi, Zhi-Ming Yuan, Xiao-Ming Yang, Shuo Shen

**Affiliations:** a Wuhan Institute of Biological Products Co. Ltd., Wuhan, People’s Republic of China; b National Engineering Technology Research Center of Combined Vaccines, Wuhan, People’s Republic of China; c Center for Biosafety Mega-Science, Chinese Academy of Sciences, CAS Key Laboratory of Special Pathogens, Wuhan Institute of Virology, Wuhan, People’s Republic of China; d National Institutes for Food and Drug Control, Beijing, People’s Republic of China; e JOINN Laboratories (Beijing), Beijing, People’s Republic of China; f Department of Medicine, University of Cambridge, Addenbrooke’s Hospital, Cambridge, UK; g China National Biotec Group Company Ltd, Beijing, People’s Republic of China

**Keywords:** SARS-CoV-2, inactivated vaccine, immunogenicity, toxicity, animal models

## Abstract

The ongoing COVID-19 pandemic is causing huge impact on health, life, and global economy, which is characterized by rapid spreading of SARS-CoV-2, high number of confirmed cases and a fatality/case rate worldwide reported by WHO. The most effective intervention measure will be to develop safe and effective vaccines to protect the population from the disease and limit the spread of the virus. An inactivated, whole virus vaccine candidate of SARS-CoV-2 has been developed by Wuhan Institute of Biological Products and Wuhan Institute of Virology. The low toxicity, immunogenicity, and immune persistence were investigated in preclinical studies using seven different species of animals. The results showed that the vaccine candidate was well tolerated and stimulated high levels of specific IgG and neutralizing antibodies. Low or no toxicity in three species of animals was also demonstrated in preclinical study of the vaccine candidate. Biochemical analysis of structural proteins and purity analysis were performed. The inactivated, whole virion vaccine was characterized with safe double-inactivation, no use of DNases and high purity. Dosages, boosting times, adjuvants, and immunization schedules were shown to be important for stimulating a strong humoral immune response in animals tested. Preliminary observation in ongoing phase I and II clinical trials of the vaccine candidate in Wuzhi County, Henan Province, showed that the vaccine is well tolerant. The results were characterized by very low proportion and low degree of side effects, high levels of neutralizing antibodies, and seroconversion. These results consistent with the results obtained from preclinical data on the safety.

## Introduction

The coronavirus disease 2019 (COVID-19) is caused by a newly emerged, zoonotic, severe acute respiratory syndrome coronavirus-2 (SARS-CoV-2). It has jumped the species barrier and was transmitted from animals to humans with a strong tendency to establish itself in human population [1–4]. The ongoing COVID-19 pandemic imposes massive public health and economic burdens worldwide. The SARS-CoV-2 is closely related to SARS-CoV. They share 69.5% of the nucleotide sequence identity and use the same receptor for entry into cells. Vaccine candidates against COVID-19 are urgently needed to protect the general population, in particular high-risk groups such as the elderly and health care personnel. Strategies are required for rapid vaccine development, production at large scale, and widespread distribution worldwide. In doing so, critical requirements on vaccine safety and efficacy must be followed as recommended by the WHO and prescribed by national regulatory authorities [5,6].

SARS-CoV-2 is a member of the genus *Betacoronavirus* in the family *Coronaviridae*. It belongs to the same *Betacoronavirus* lineage as the SARS-CoV and also uses the angiotensin converting enzyme 2 (ACE2) as receptor [3,7,8]. The virion is approximately 100–150 nm in diameter [9]. Spike glycoprotein (S), the membrane protein (M), accessory 3a protein, and the envelope protein (E) are located on the surface of virion, and the nucleocapsid protein (N) binds to the viral RNA in helical symmetry, forming the ribonucleocapsid inside the viral particle. Three S protein monomers form a homotrimer, which is the major antigen eliciting neutralizing antibodies, and thus a major target for vaccine development. Several strategies have been employed to express the S or truncated S protein, such as mRNA/DNA vaccines, adenovirus – and influenza-virus vector-based vaccines, and subunit vaccines based on previous experiences in SARS-CoV and SARS-CoV-2 vaccine developments [10–16]. An alternative strategy is to take the advantage of a mature platform and to develop inactivated, whole virus particle-based vaccines.

The safety and effectiveness of four inactivated, full particle vaccines have been evaluated in immunization-challenge model of Rhesus monkeys and hACE2 expressing mice, including our vaccine candidate in preclinical studies and before phase I/II clinical trials (17–20). The overall results of these experiments showed the increased neutralizing antibody (NtAb) titres, reduction of virus loads, and no antibody dependent enhancement (ADE) upon challenge with wildtype viruses. The first inactivated SARS-CoV-2 vaccine, started phase I and II clinical trial on the 12th and 24th of April, 2020 shortly after the isolation of SARS-CoV-2 on the 5th of January [3,21] and entered phase III trials in the middle of June 2020.

In this report, a β-propiolactone double-inactivated, full virion vaccine against SARS-CoV-2, 2019-nCoV (Vero), was evaluated in seven species of experimental animals. The study focused on the immunogenicity, toxicity, the effect of adjuvant, routes and dose of administration, immunization schedule, immune persistence, consistency of vaccine preparation, and relative potency in stimulating neutralizing antibodies of this vaccine candidate. The results obtained from seven animal species showed a strong potency in stimulating humoral response of our vaccine candidate without causing toxicity in animals. Also, consistent and high performance of different bulks and lots of vaccine preparations have been determined. The safety profiles and immunogenicity in preclinical study described in this report is consistent with the outcome of premilitary phase I/II results (21).

## Materials and methods

### Ethical approval

The animal protocol was approved by the Animal Ethics Committee of the Wuhan Institute of Biological Products (WIBP) (WIBP-AII382020001). All experiments were performed in accordance with the relevant guidelines and regulations in place in China [22]. Clinical samples were collected from patients with signed consensus according to ChiCTR2000030046.

### Cells and viruses

Vero E6 (ATCC) and Vero (WIBP cell bank) cells were maintained in complete DMEM medium (Gibco), supplemented with newborn calf serum (NCS, 10%), streptomycin (0.1 mg/ml), and penicillin (100 units/ml) (Gibco). Cells were infected at a multiplicity of infection (MOI) of 0.1–0.001 plaque forming unit (PFU) per cell. Viruses were cultured in maintenance medium (DMEM) supplemented and 1% Antibiotic-Antimycotic (Gibco, 15240-062) in the absence of NCS. The isolation and the complete genome sequence of the WIV-04 strain (IVCAS 6.7512) of SARS-CoV-2 was as described previously [3].

### Titration of viruses and antisera

The virus stocks were titrated 6–10 times by PFU assays in Vero E6 or Vero cells. Titres were expressed as PFU/ml. Antisera were inactivated at 56°C for 30 min before use. The antisera were diluted 20-fold first, and then 4-fold serial dilutions were prepared in maintenance medium. The virus suspension (0.25 ml, 600 PFU/ml) was mixed with equal volume of antiserum at desirable dilution and incubated for 1 h. The mixture was added to monolayer cells in 12-well plates and incubated for 1 h. Following removal of the mixture, 2 ml of maintenance medium containing 0.9% of methylcellulose (Sigma) were added to each well. The plates were incubated in a 5% CO_2_-air incubator at 37°C for 3–4 days. The neutralizing titre was calculated as reciprocal of the highest antiserum dilution suppressing 50% of plaque forming. Plaque reduction NtAb titre (PRNT, 95% CI, challenge viruses used: 30–300 PFU/well) was calculated as the “inhibitor vs normalized response (Variable slope)” model in the GraphPad Prism 8.0 software.

### Analysis of viral structural proteins and virions

The purified vaccine was concentrated by using Amicon Ultra-15 centrifuge tube (Ultracel-3 kDa). After centrifugation at 3000 *g* at 4°C for 3 h**,** the samples were used for poly-acrylamide gel electrophoresis (PAGE) and Western blotting (WB) analysis using antibodies directed against the virions. Total proteins were separated on an 8% or 4–20% gradient poly-acrylamide gel. The wet-transfer for 3–4 h was performed to transfer proteins to NC membrane. The anti-SARS-CoV-2 antiserum was raised against purified virions in rabbits using Freund complete/incomplete adjuvants at a dose of 25 µg/injection via s.c. route. Antiserum was obtained after 4 times injections and WB was performed according to the procedure previously described [23]. Purified virus particles were examined by transmission electron microscopy (TEM) following negative staining with methylamine tungstate.

### Detection of total specific antibodies in antisera with ELISA

The 96-well plates were coated with inactivated, purified virions at a concentration of 1 μg/ml (100 μl/well) in 0.05 M Carbonate Buffer Solution, pH 9.6, at 4°C overnight. The plates were blocked with 200 μl of 0.01 M PBST-1% bovine serum albumin (BSA) per well. Following incubation at 37°C for 1 h, 100 μl of serum samples, in a 10-fold or 2-fold series of dilutions, were added per well and incubated for 1 h. Then, 10 μl of HRP-conjugated antibodies against IgG (Boster Biological Technology) of different hosts at a dilution of 1:2000–5000 were added followed by incubation for 1 h. The substrates of 1.25 mM 3′, 3′, 5, 5′-Tetramethyl benzidine and 6.52 mM urea hydrogen peroxide were added to each well (50 μl each) and incubated for 30 min. The reaction stop solution was added, OD_450_ and OD_630_ values were obtained, and higher than cutoff value of 0.15 was considered as positive. Between each step, the wells were washed five times with PBST.

### Immunization of animals

Seven different animal species were used for evaluation of toxicity and immunogenicity of the vaccine candidate: Balb/c mice, SD rats (Sprague–Dawley), Wistar rats, Guinea-pigs (Hartley), Japanese white rabbits, Cynomolgus monkeys (*Macaca fascicularis*), and Rhesus monkeys (*Macaca mulatta*).

The adjuvants Aluminium hydroxide (Alum) (Croda Denmark, Cat No. 85643) and MF59 (kindly provided by Dr. Hu YQ, RM118) were used at concentrations of 0.5 mg/dose and 0.25 ml/dose, respectively, for animal immunization. Negative controls included Alum-buffer, MF59-buffer and buffer-only samples. Number of animal in each group, immunization routes, times of boosting and immunization intervals, doses per injection, age and gender of animals were described in individual experiments. Bleeding times and interval of animal immunization were also described in individual experiments.

High, medium, and low doses are 25, 5, and 1 μg, respectively, or are indicated in individual experiments. Total protein concentration and purity of the vaccine were determined by standard methods of BCA, PAGE, and WB assays [23]. The percentage of virion is calculated by formula, Total viral protein mass = Total concentration of proteins × % of purity. The specific antigens of the S glycoproteins are determined by the mass of virions times percent of the S proteins. If non-specific neutralizing titres in the sera of the control animals were below 20, the values were assigned as 10 for convenience of presentation in the figures.

## Statistical analysis

Prism 8.0 software was used to calculate Geometric Mean Titres (GMT) of neutralizing antibody titres, the mean and standard deviation (SD) for OD values of total IgG. One way-ANOVA with Sidak’s multiple comparisons tests were used to determine significance of differences and to determine significant correlation of different antibody titre measurements.

## Results

### Virus isolation, adaptation, and characterization

One of seven viral RNA positive specimens from COVID-19 patients, hospitalized in Jinyintan Hospital at the City of Wuhan, was used to isolate viruses in Vero E6 cells in Biosafety Level 3 laboratory at the WIV [3]. The WIV-04 strain was well adapted to growth to high titres in Vero E6 cells and was adapted to grow in Vero cells preserved for vaccine production at WIBP. The Vero cell line has been qualified and permitted for production of human vaccines by Chinese regulatory authorities [5].

The virus passage 7 of WIV-04 strain was further purified three times through growth by limiting dilution method. A purified virus clone WIV04-1 was chosen and passaged, based on its high titre and yield of virus particles. Virus primary seed, master seed and working seed lots were established (namely, passage 11, 12, and 13) and characterized following the guidelines by Chinese regulatory authorities for the production of viral vaccines [5]. The genetic stability of viruses was analysed by sequencing of the full-length genomes of different passages. No nucleotide changes in the complete genome were identified for the purified clone during the passaging. The adventitious agents were examined and excluded by both deep sequencing and standard neutralization assay with high titre and specific antibodies against SARS-CoV-2. Other conventional methods were also used for characterization of viruses required by Chinese regulatory authorities [5].

### Preparation of the inactivated SARS-CoV-2 vaccine

The developed processes of the vaccine preparation and quality control methods were submitted for patent applications. Briefly, the processes included the cell culture and virus propagation, harvesting, β-propiolactone-inactivation ([2]1:4000 (v/v) at 2–8°C for 48 h, followed by cell debris clarification, ultrafiltration, 2nd *β*-propiolactone-inactivation, gel-chromatography, ion-exchange chromatography, sterile filtration. Formulation with buffer and aluminium hydroxide (Alum), filling, packaging, and labelling were performed. Inactivation was validated by passaging the treated samples 3 generations without appearance of CPE. The in-process quality controls were established and applied. Production was performed in good manufacturing practice (GMP) manufacturing facilities and well documented in detail. The preliminary stability of the finished products was also tested at different temperatures. The persistency of quality and quantity of each bulk and lot of preparations was determined. The residues of host Vero cell proteins, DNAs, and additives in cell culture were detected under permissive levels following the guidelines of the national regulation authority by dot-blotting, quantitative real-time PCR, and other applicable methods.

**Figure 1.**
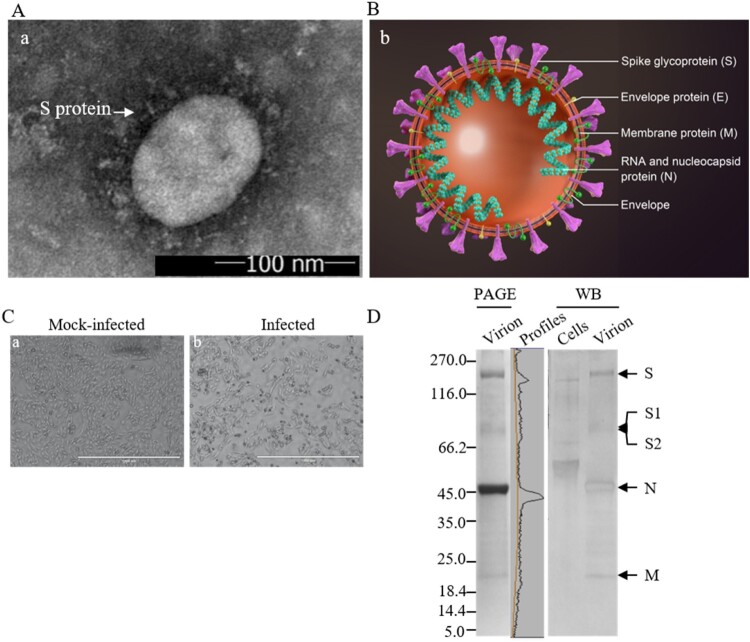
Characterization of vaccine candidate. (A) The negatively-stained, inactivated virion of SARS-CoV-2 visualized by TEM. The arrowhead indicates the spike (S) glycoproteins on the viral envelope. Scale bar = 100 nm; magnification, ×50,000. (B) Schematic model of SARS-CoV-2 particle. The major structural proteins S, M, and E, are located in the lipid envelope, enclosing the viral RNA-N protein complex (ribonucleoprotein). (C) Vero E6 cells mock-infected or infected with SARS-CoV-2 at a MOI of 0.01 and observed at 48 h post-infection by light microscopy. Scale bar = 1000 μm. (D) Analysis of proteins of the purified, inactivated SARS-CoV-2 by PAGE and Western blotting. Viral proteins were separated by 4–20% gradient PAGE, and Western blotting was performed using rabbit anti-virion antibody (1:500), identifying the structural proteins S, N, and M as indicated. Convalescent sera collected from patients of COVID-19 were used for characterization of immune responses against the viral proteins in virions. Mock-infected cell lysate was used as a control. PAGE gel was scanned by using Quantity One software. Molecular weight markers (in kDa) are indicated on the left. Viral structural proteins are indicated on the right.

Three to four days post-infection, when more than 80% of the infected Vero cells were round-up and detached from the surface of culture vessels, the supernatant was harvested for vaccine preparation. Negatively-stained SARS-CoV-2 showed that the virions were spherical and pleomorphic under TEM at a size of 100–150 nm in dimeters (Figure 1(A,B)). A typical cytopathic effect (CPE) is shown in Figure 1(C). The infectivity titres of harvested virus were between 7.5 and 8.0 Lg PFU/ml. The spike proteins are clearly visible from EM images on the surface of the envelope, suggesting that the native conformation of homotrimers of the spikes remained intact. The major neutralizing antigens on the viral envelope were well preserved through the production processes. One of the reasons is that the inactivation with *β*-propiolactone was performed at low temperature 2–8°C, acting on the viral RNA not the proteins.

### Structural protein profiles of the purified virions

The inactivated, purified virions were analysed by PAGE and Western Blotting using the rabbit anti-virion antiserum and convalescent sera collected from a recovered COVID-19 patient (Figure 1(D)). The full-length S and subunits S1and S2 after cleavage by Furin-like proteases were detected as predicted [24,25]. The 3a, N, and M proteins were observed and confirmed. The structural protein E with a calculated MW of 8.4 kDa was not detectable, possibly due to its small size, low molar ratio among the structural proteins or weak reactivity with the anti-virion antiserum. The deduced molecular weights of the S, N, 3a, and M proteins are 142.1, 45.6, 29, and 25 kDa, respectively. As the S proteins are heavily glycosylated [26] and other proteins are modified, the apparent molecular weights of the full-length S, S1 and S2 and N, 3a, M proteins are approximately 200, 115, 90, 55, 35, and 26 kDa, respectively. The virion may contain degraded viral proteins and other accessory proteins including the 3a [27,28], which needs to be further clarified in detail using antibodies against the accessory proteins. The protein profiles of the inactivated virions on the gels were scanned and the relative quantity of the S, S1/S2, N, M, and 3a was 22.6, 10.6, 51.3, 8.9, and 2.5%, respectively. The purity of the vaccine was calculated to be greater than 95.9% of the total proteins, and the proportion of the neutralizing antigen S was 33% of the viral proteins based on PAGE and WB analysis. The proportion of the full-length S to S1/S2 was approximately 2 to 1. The cleaved S1 and S2 remained to be part of the S trimers on the viral envelope.

### Immunogenicity and toxicity in animals

Seven species of animals were used for analysis of immunogenicity. Animals used were pathogen free, with the exception of rabbits and monkeys, raised at the WIBP or purchased from other sources. Different formulations and immunization schedules were assessed for immunogenicity, dynamics of specific antibody responses, antibody persistency, and toxicity in animals. The main correlates of potency of the vaccine candidate were the amounts of total virus-specific IgG and NtAb. The NtAb titres were compared with those observed in acute and convalescent sera from patients and patients recovered of COVID-19, providing a basis to the dosage of the vaccine candidate and immunization schedules at the beginning of the clinical trials. Toxicity of the vaccine candidate was also evaluated in three species of animals.

### Evaluation of inactivated vaccine in Balb/c mice

#### Priming and boosting schedule with 7-day interval

**Figure 2.**
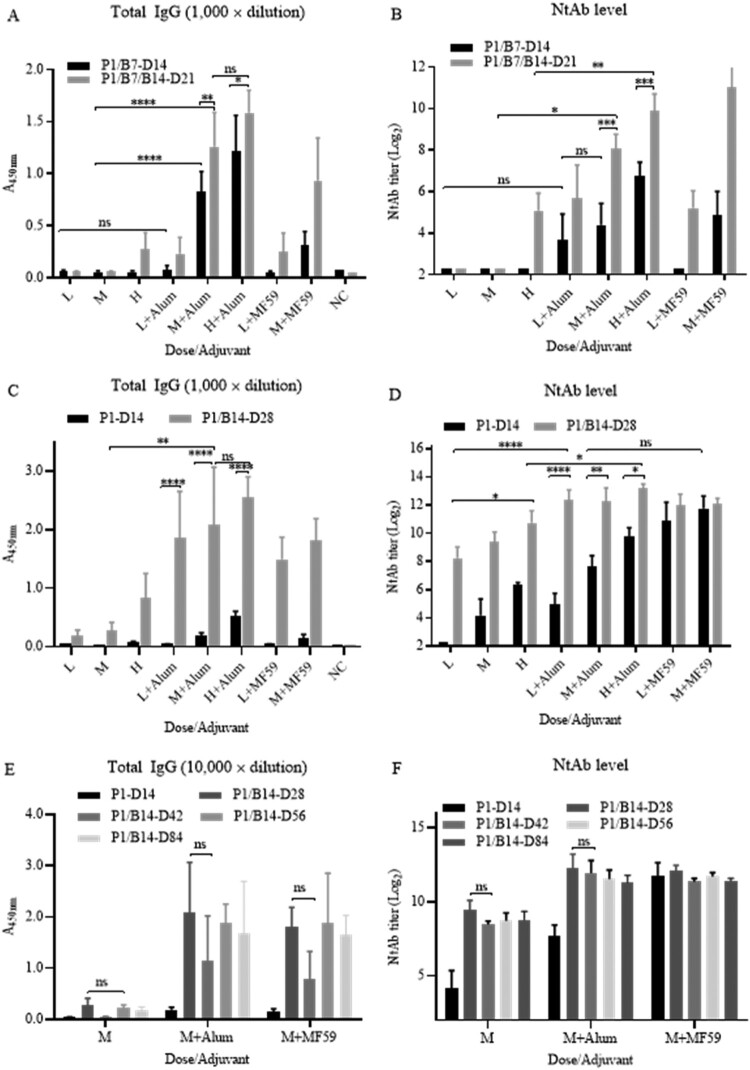
Effects of adjuvants, doses, boosting times, and intervals on immunogenicity. Immunization was performed in 6–8 weeks old, female Balb/c mice (*n* = 5) via i.p. route. Low, medium, and high doses (1, 5, 25 μg/mouse) of the vaccine candidate were inoculated with or without adjuvants Alum and MF59 as indicated. (A) and (B) P1/B7-D14: priming on day 1, boosting on day 7, and bleeding on 14; P1/B7/B14-D21: priming on day 1, boosting days 7 and 14, bleeding on day 21. (C) and (D) P1-D14: priming on day 1 and bleeding on 14; P1/B14-D28: priming on day 1, boosting on day 14, bleeding on day 28. The specific IgG titres were expressed with the value of A450 nm, while the sera were 1000-fold-diluted. The column sizes and error bars represent GMT ± SE values. Amounts of virus-specific IgG against virion (A, C) and NtAb titres (B, D) levels were detected as indicated. (E) and (F) Specific antibody persistence post-priming o r/and boosting in Balb/c mice. The conditions of experiments were the same as the previous experiments described in the legend of Figure 2(A,B), but only the medium dose was used. The schedules were priming only and priming-boosting at 14 d intervals. The bleedings were carried out on days 14, 28, 42, and 56. Total specific IgG amounts against virions and NtAb titres were determined in sera. The specific IgG titres were expressed as the value of A450 nm, while the sera were 10,000-fold-diluted. The column sizes and error bars represent GMT ± SE values. The Sidak’s multiple comparisons were used to determine significance of differences, *, **, ***, ****, ns, indicating *P* < 0.05, *P* < 0.01, *P* < 0.001, *P* < 0.0001 and no significant statistical difference, respectively.

As shown in Figure 2, levels of total specific IgG (Figure 2(A)) against virion was correlated with NtAb titres (Figure 2(B)). The antibodies were detected 7 days post-priming at a high dose without adjuvants and at low dose with adjuvants, compared the specific IgG and NtAb titres at different bleeding time.

Adjuvant enhancement of humoral immunogenicity was significant. Without adjuvants, specific IgG and NtAb titres were only detected in mice under conditions of high dose and boosting twice on day 7 and 14 (P1/-B7/B14-D21, high dose). In contrast, antibody levels increased significantly at low, medium, and high doses when adjuvants were used. There was no significant difference between Alum and MF59 at low dose (*P > *0.05). Alum was equally as strong as adjuvant MF59 and slightly higher at medium dose when Alum was used.

Both IgG and NtAb titres increased in a dose-dependent manner when levels elicited by doses (1, 5, and 25 µg) were compared. Titres of IgG and NtAb were higher when the mice were boosted twice instead of once, also demonstrating a specific dose-dependent effect.

#### Priming and boosting schedule with 14-day interval

Next, immunization schedule with 14 days interval was tested (Figure 2(C,D)), The priming and boosting was performed on day 1 and 14, respectively, and bleeding were performed on day 14 and day 28. NtAb titres reached high levels with both adjuvants at low, medium and high doses on day 14 post-priming and day 14 post-boosting, even without adjuvants. The levels of total specific IgG were proportional to those of the NtAb titres. Schedule with a 14 days interval clearly induced much higher levels of total IgG and NtAb than those of the schedule with a 7 days interval (Figure 2(A,B)) in Balb/c mice.

#### Immune persistency post-priming or/and boosting in Balb/c mice

The persistency of specific antibodies directed against the candidate vaccine was investigated in Balb/c mice. Groups of mice were immunized with a 14 days interval at the medium dose (5 μg/mouse) using both adjuvants Alum and MF59. As shown in Figure 2(E,F), post-priming on day 1 and boosting on day 14, the NtAb titres reached high levels on day 28 and remains at the same levels to days 42, 56, and 84, correlating with the increases of the total specific IgG. This also demonstrated that boosting once at least was needed for a high total-specific IgG and NtAb responses in mice. The preliminary results of immune persistency are promising, and the mice are kept for monitoring pf immune persistency in longer time.

### Humoral immunogenicity vaccine candidate in Wistar rats via 3 different routes of administration

**Figure 3.**
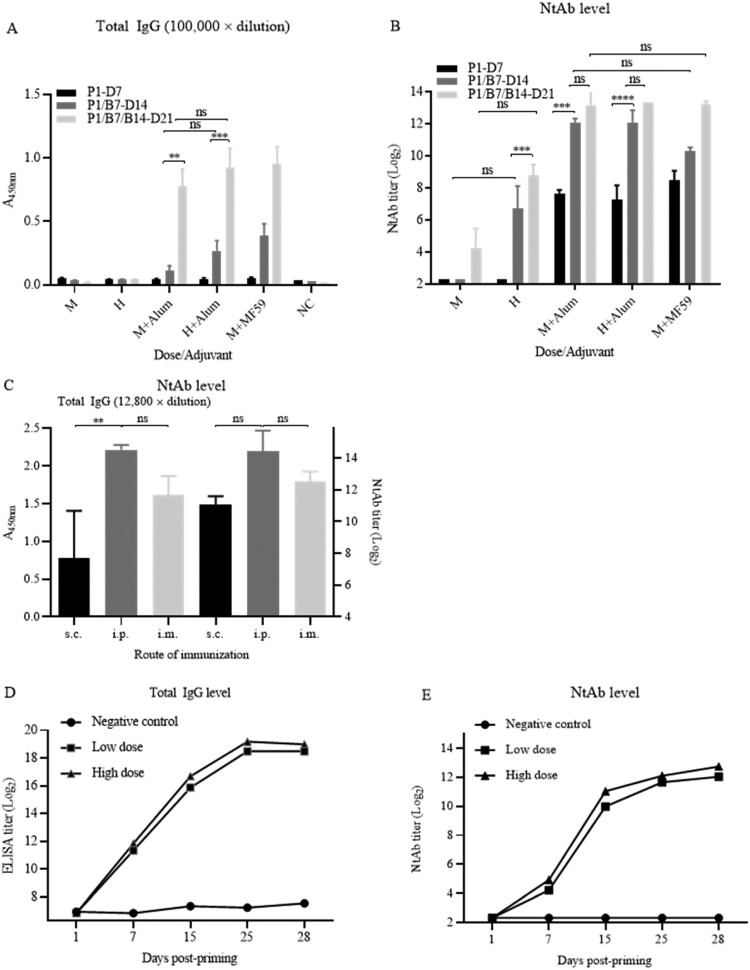
Humoral immunogenicity in rats. (A) and (B) Humoral immunogenicity in Wistar rats. Immunization was performed in groups of female Wistar rats (body weight, 175–200 g; *n* = 5) via i.m. route. High and medium doses (25 and 5 μg/rat) of the vaccine candidate were inoculated with or without adjuvants Alum and MF59 at the schedule of P1/B7-D14 as indicated. P1-D7: priming on day 1 and bleeding on day 7; P1/B7-D14: priming on day 1, boosting day 7, and bleeding on day 14; P1/B7/B14-D21: priming on day 1, boosting on day 7 and 14, bleeding on day 21. Total specific IgG against virion and NtAb titres were detected as indicated. The specific IgG titres were expressed with the value of A_450nm_ while the sera were in 100,000-fold dilutions. The column sizes and error bars represent GMT ± SE values. The Sidak’s multiple comparisons were used to determine significance of difference, with *, **, ***, ****, ns, indicating *P* < 0.05, *P* < 0.01, *P* < 0.001, *P* < 0.0001 and no significant difference, respectively. (C) Immunogenicity in Wistar rats via different routes. Three groups of (female) Wistar rats (*n* = 5) were primed and boosted via s.c., i.p., and i.m. routes at a dose of 5 μg/rat on an interval of 7 days. Bleedings were performed on day 7 post-boosting and total IgG and NtAb titres were determined. (D) and (E) Humoral immunogenicity in Sprague–Dawley (SD) rats. Groups of male and female SD rats (*n* = 5) were primed and boosted via i.m. route at low and high doses (5 and 15 μg/rat) of the vaccine on days 1, 7, and 15. Bleedings were performed on the same days and on days 25 and 28. The rats in control group were inoculated with buffer only. Total virus-specific IgG and NtAb titres were determined.

First, immunogenicity of the vaccine candidate was tested in Wistar rats with medium and high doses (5 and 25 μg) with both adjuvants at an immunization interval of 7 days. The results showed that the increases of total specific IgG and NtAb titres depends on dose, boosting interval and adjuvant for stimulating strong humoral immune responses (Figure 3(A,B)).

Next, 3 groups of Wistar rats (*n* = 3) were immunized with 5 μg at an interval of 7 days through three routes, intraperitoneal, intramuscular and subcutaneous injections. There were no differences in NtAb titres among the 3 groups via different routes (Figure 3(C)). The total IgG level via s.c. route was lower than those via i.p. and i.m. routes (*P *< 0.01).

### Evaluation of humoral immunogenicity and toxicity of vaccine candidate in Sprague–Dawley (SD) rats

#### Immunogenicity

Seven days post-priming, 50% (5/10) and 60% (6/10) of rats in medium and high doses (5 and 15 μg per rat) were seroconverted. GMTs of NtAb increased to 1009 and 2094 seven days after the 1st boosting while GMTs of NtAb increased further at low and high doses following the 2nd boosting, reaching the peak (GMTs 3200 and 4361) 10 days post-2nd boosting in a 7 days interval (Figure 3(D,E)). The NtAb titres increased continually after the second boosting, demonstrating specific immunogenicity of the vaccine candidate. The antibody levels elicited by the high dose were higher than that elicited by the low dose, indicating the increase of NtAb in a dose-dependent manner in SD rats (*P *< 0.01). The increases of total specific IgG correlated with those of NtAbs.

#### Toxicity

A total of 150 rats (75 animals/gender) were assigned to 7 groups, of which 120 rats were allocated to Groups 1, to 4 (15 animals/gender/group) for the toxicity study, and 30 rats were allocated to Groups 5, to 7 (5 animals/gender/group) for the TK study. Animals were administered sodium chloride injection for the negative group, or adjuvant control for adjuvant control group, the dose volume was 0.5 ml/animal, or test article at dosage of 1 and 3 dose/animal for the low- and high-dose test groups via intramuscular injection, respectively. Under the conditions of this study, no obvious systemic toxicity was observed in the animals at the dosage of 1 and 3 doses/animal during the period of administration and the end of 2-week recovery period. It is considered that the no observed adverse effect level was 3 doses/animal.

### Humoral immunogenicity and active systemic anaphylaxis of vaccine candidate in Guinea pigs

#### Immunogenicity

A group of Hartley guinea pigs (*n* = 5) was primed and boosted twice at a dose of 5 μg of the vaccine candidate via s.c. route at an interval of 7 days. Bleeding was performed on day 7, 14, and 21 post-priming and NtAb titres were determined as shown in Table 1. NtAb titres increased 7 days post-priming and post-2nd boosting to 5120 and 10,240, respectively. The specific immune response was dose-dependent as the differences in NtAb titres between priming and the 1st and 2nd boosting were statistically significant (*P *< 0.001, *P *< 0.0001).

**Table 1.** NtAb titres (GMT) of antisera in Hartley guinea pigs, rabbits, and Rhesus monkeys.

Schedule	Guinea pigs (Dose μg)	Rabbits (Dose μg)			Rhesus monkeys (Dose μg)		
	(5)	J-1 (25)	J-2 (25)	J-5 (5)	Controls	(5)	(25)
P1-D7	40	NA		NA	NA	NA	NA
	40				NA	NA	NA
	5				NA	NA	NA
P1-D14			66				
P1/B7-D14	298	226		NA	150	730	1510
	822				90	1124	2474
	430					1932	574
P1/B14-D21			5120				
P1/B7/B14-D21	5120	5120		800		1520	3028
	5120				124	1146	3472
	10,240				110	2776	2776
	5120						
P1/B14/B28-D42			2528				
P1/B7/B14/B21-D35		2262					

Note: NA, not assayed. Female Guinea pigs, 250–300 g. P, priming day; B, boosting day; D, bleeding day. Japanese white rabbit, 2.2–2.5 kg, antigens formulated with Freund complete/incomplete adjuvants. J-5 immunized with vaccine containing 5 μg antigen formulated with Alum adjuvant. J-1 and J-2 priming on day 1, boosting days 7, 14, and 21. Bleeding, 7 days post-immunization. J-2 priming on day 1, boosting days 14 and 28. Bleeding 14 days post-immunization. Rhesus monkeys, 4 males and 4 females, 5.9–10.9 kg/animal. All animals were immunized via s.c. route.

#### Safety

A total of 36 male guinea pigs were randomly assigned to 4 groups (9 animals/group): negative control group (sodium chloride injection), positive control group (human albumin, 20 mg/animal for sensitization and 40 mg/animal for challenge), low and high dose of test group (0.1 dose/animal for sensitization, 1 dose/animal for challenge) by intramuscular injection. No mortality or moribundity was noted and no abnormal reaction was noted in clinical observation and body weight in all animals during the study period of 4 weeks.

### Humoral immunogenicity of vaccine candidate in Japanese white rabbits

Three Japanese white rabbits (2.2–2.5 kg) were immunized with a dose of 5 μg (J5) and 25 μg (J1 and J2) formulated with Alum and Freund complete (priming)/incomplete adjuvants (boosting) via s.c. route. The NtAb titres increased following the post-1st or 2nd boosting, showing variation between the rabbits (Table 1). None of the rabbits showed adverse effects following the inoculation of the vaccine candidate.

### Humoral immunogenicity of vaccine candidate in Cynomolgus and Rhesus monkeys

#### Cynomolgus monkeys (*Macaca fascicularis*)

**Figure 4.**
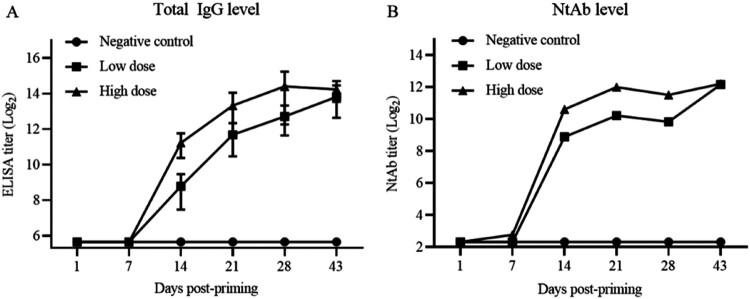
Humoral immunogenicity of vaccine candidate in Cynomolgus monkeys (*Macaca fascicularis*). Three groups of Cynomolgus monkeys (*n* = 10) were inoculated via i.m. route at low and high doses (5 and 20 μg/monkey) or mock-inoculated with Alum only on days 1, 7, 14, and 28. Bleeding was performed on day 7, 14, 21, 43 and total IgG and NtAb levels were determined. Error bars represent GMT ± SE values.

*A. Humoral immunogenicity of vaccine candidate*. Immunogenicity of vaccine candidate in Cynomolgus monkeys was determined and the results were shown in Figure 4. Seven days post-priming, the antibody levels in the sera of inoculated animals were not different from those of control animals. Seven days post-1st boosting, total IgG titres increased to 237 and 2111, respectively, for monkeys of low- and high-dose groups. The IgG levels continuously increased to 2786 and 8445, respectively, in both low- and high-dose group post-2nd boosting. The immune responses showed the boosting effect and specific dose-dependent effect, comparing with negative results for control animals. The levels of NtAb were also determined and correlated with those of the total specific IgG levels.

*B. 6-Week toxicity study of vaccine candidate*. A total of 40 cynomolgus monkeys were randomly assigned to 4 groups (5 animals/gender/group) including the saline control, adjuvant control, low (5 μg, 1 dose), and high dose (20 μg, 4 doses) groups via i.m. route, once weekly or every two weeks for 4 weeks on days 1, 8, 15, and 29.

During the study, no differences in body weight, body temperature, electrocardiogram (ECG), blood pressure, oxyhaemoglobin saturation, ophthalmology, clinical pathology (haematology, coagulation, clinical chemistry, urine analysis) were observed or detected in animals inoculated with the vaccine, compared with control groups. There are no differences between vaccine-inoculated animals and control animals in numbers of lymphocyte subsets (CD3+, CD3+CD4+, CD3+CD8+, CD3+CD4+/CD3+CD8+, and CD20+), and cytokine profiles (IL-2, IL-4, IL-5, IL-6, TNF-*α*, IFN-*γ*), C-relative protein, complements (C3, C4). The specific IgG antibody and the neutralizing antibody against the vaccine candidate were detected only in vaccine-inoculated animals but not in control animals.

Taken together, no obvious systemic toxicity was observed in any animals during the dose period and at the end of 2-week recovery period. The no observed adverse effect level may be more than 4 dose/animal. Irritation reactions related to aluminium adjuvant were noted in injection sites. The specific IgG antibody and the neutralizing antibody activity were detected positive, and no immunotoxicity was observed in animals.

#### Rhesus monkeys (*Macaca mulatta*)

Three groups of Rhesus monkeys were inoculated or mock-inoculated with low and high doses of the vaccine (5 and 25 μg/animal) or adjuvant-only via i.m. route on days 1 and 14. The NtAb titres were detected on days 14 and 21 as shown in Table 1. Dose-depending and boosting effects were observed. Immune responses were specific compared with that of the mock-immunized monkeys.

### Consistency of the production process and potency of the vaccine candidate

**Figure 5.**
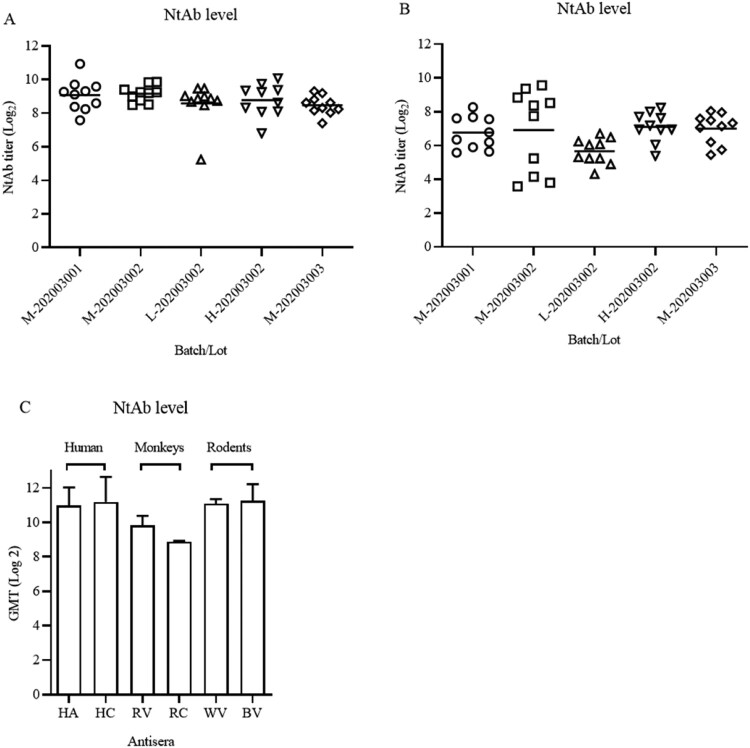
Persistence of potency between different bucks in Balb/c mice, Wistar rats and comparison with NtAb titres of human sera. (A) and (B) Groups of Balb/c female mice (i.p., *n* = 10) and Wistar rats (i.m., *n* = 10) were inoculated with candidate vaccines of 5 lots from 3 different bulks of production at low, medium, and high doses (2.5, 5, and 10 μg/rat) of the vaccine on day 1. Bleedings were performed on day 14 and total IgG and NtAb levels were determined. Circles, triangles, and squares represent individual animals. Average titres are indicated with lines. (C) Comparison of immune responses in immunized animals and patient sera. HA, HC: Human acute and convalescent sera from 9 patients and 7 recovered patients of COVID-19, respectively. RV, RC: antisera and convalescent sera from immunized (5 µg) and infected monkeys. WV, BV: Wistar rat and Balb/c antisera from virion-immunized animals at a dose of 5 µg.

The consistency of production process between bulks and lots was studied in Balb/c mice and Wistar rats. The NtAb titres were determined using doses of 2.5, 5, and 10 μg/animal, providing one of the critical quality control measures in the vaccine production. As shown in Figure 5(A), NtAb GMTs were 539, 570, 385, 438, and 356 for 5 lots in sera of immunized Balb/c mice. The GMTs were similar among the animals in each medium dose groups (5 μg), indicating the consistency of the production process with regard to this parameter. NtAb GMT in Wistar rats were lower than those Balb/c mice (Figure 5(B)).

Finally, NtAb GMTs of human acute and convalescent sera (from 9 and 7 patients, respectively) were compared with those of antisera in immunized Balb/c mice, rats, monkeys (5 μg/animal, antisera from infected and vaccinated monkeys). As shown in Figure 5(C), NtAb titres in animals immunized with 5 μg were similar with those of human acute and convalescent sera at the high end of the total numbers of 153 convalescent sera (data not shown).

## Discussion

The safety and efficacy are the major concerns in the development of vaccines against COVID-19 based on the previous research on the SARS-CoV and MERS-CoV. Our preclinical research on the inactivated vaccine candidate has been focused on the evaluation of these issues in as many species of animals as possible. The results showed that the vaccine candidate strongly stimulated immune responses and presented no toxicity in animals tested. Biochemical analysis showed that a portion of the spike glycoprotein was cleaved into subunits S1 and S2 and the three forms of S were bearing the neutralizing epitopes of SARS-CoV-2.

The spike glycoprotein of coronaviruses is a class I viral fusion protein. The S protein is a co-translationally N-glycosylated in the ER, oligomerized if folded properly with the help of host proteins, and associated with the M protein in pre-Golgi and ER-Golgi compartment membranes [29–31]. The S protein of SARS-CoV-2 forms homotrimer and may be cleaved by host furin-like proteases into a S1 subunit for receptor binding (ACE2) and a S2 subunit for membrane fusion [3]. Both receptor binding domain and viral-cellular membrane fusion domains, and probably other regions, carry neutralizing antigen epitopes [32]. The homotrimers of the S proteins in the inactivated virions of SARS-CoV-2, assembled in mammalian cells, likely maintain their native structure, conformation, and neutralizing epitopes. These explained its excellent humoral immunogenicity in animals tested and in phase I/II clinical trials [21]. In this study, the cleavage of the monomeric S into S1 and S2 was also confirmed. Further characterization of the folding, glycosylation, maturation, trimer-formation, and cleavage in Vero cells are under way. The relationship between these post-translational modifications and the functions of the S protein, such as virus entry of cells, immunogenicity warrants further investigations.

The S protein is a membrane-bound protein with a relatively short transmembrane domain. The changes in the amino acid sequence and temperature may affect the stability [33]. The process development of vaccine production has been focused on the selection of a vaccine strain, which is stable and produce high titre and a high yield of viral particles. It is also very important to reduce the shearing force during procedures to keep the native form of the major neutralizing antigen in the production processes of inactivated SARS-CoV-2 vaccine.

One critical aspect of vaccine against COVID-19 is the immune persistency. Little is known about the memory B-cell responses against SARS-CoV and MERS-CoV, apart from a recent study which demonstrated the persistence of anti-MERS-CoV antibodies in MERS survivors for up to 34 months [34]. On the other hand, antibody responses against another closely related coronavirus, SARS-CoV, were not persistent, since a 6-year follow-up study did not detect memory B-cell responses in SARS survivors [35]. Immune senescence is common in the elderly, who are one of the main targets of protection by a SARS-CoV-2 vaccine. Dosages and immune schedule study are very important in animals and in the high-risk population. In this study, the preliminary results in mice and rats showed that the NtAb titres reached the peak post-priming and boosting in an interval of 14 days for 84 days at least. The mice and rats have been injected twice or three times at a dose of 5 µg/mouse and NtAb levels remain high for 6 months (Unpublished data). Longer interval between the priming and boosting increased the NtAb levels in animals, consistent with the preliminary results of phase I/II clinical trials [21]. Immune persistency will warrant further study by following up the volunteers in phase I and II clinical trials to test the NtAb titres over a long-period of time or donors of convalescence sera for plasma treatment of patients of COVID-19 [36].

One of main concerns is whether the SARS-CoV-2 candidate vaccine can cause ADE and might develop severe pathological changes in the lung or liver [37,38] when vaccinated individuals after being exposed to attack by wildtype virus. It is a controversial issue because the severe side effect of ADE has not been observed generally by other researchers of SARS-CoV vaccine development [39] and in the preclinical study of SARS-CoV-2 study [17–20]. For this vaccine, efficacy and safety were investigated in both Rhesus monkeys and ACE2 Tg mice (unpublished data). The results demonstrated that following vaccination in monkeys and transfer of specific neutralizing serum in ACE2 mice, the animals were protected from infection post-challenge with wildtype SARS-CoV-2 and no ADE was observed. Furthermore, ACE2-transcently expressed mice were used for this vaccine to provide critical evidence of safety and *in vivo* protection of the vaccinated animals [20]. Following both active immunization of the vaccine and passive immunization of antibody, the ACE2-expressing mice were protected from attack of challenging SARS-CoV-2 and no adverse reactions were observed [20]. Toxicity study was also performed in SD rats and Cynomolgus monkeys and the results would support immunization of the elderly at a high dose and boosting two times if needed.

The reported, overall results showed that NtAb titres increase, virus loads reduced and no ADE was observed in immunization of inactivated vaccine and challenging animal models [17–20]. The first inactivated SARS-CoV-2 vaccine, developed by our joined teams, started phase I and II on the 12th and 24th of April, 2020 shortly after the isolation of SARS-CoV-2 on the 5th of January 2020. Preliminary observation showed the very low degree and low proportion side effects. The high levels of NtAb and high seroconversion of NtAb of vaccine candidate is particularly promising [21]. The phase III clinical trials have been performed in multi-centres in four countries cautiously, considering potential severe adverse reactions. The phase III study involved more than 10 thousand of volunteers in four countries and no severe adverse reactions have been reported at the writing of this report.

In conclusion, study on the safety and immunogenicity of the inactivated, whole virus vaccine showed that in animals used, the vaccine was well tolerance and stimulated strong humoral immune response. These results were supporting clinical trials of the vaccine candidate especially considering GMT of neutralizing antibodies as high as those of convalescent sera. The safety, immunogenicity, and efficacy need to be investigated in clinical trials, especially the safety when the vaccinated volunteers are exposed to wildtype SARS-CoV-2.
